# Construction of a nomogram with IrAE and clinic character to predict the survival of advanced G/GEJ adenocarcinoma patients undergoing anti-PD-1 treatment

**DOI:** 10.3389/fimmu.2024.1432281

**Published:** 2024-07-24

**Authors:** Han Wang, Jinhua Chen, Wei Gao, Yilan Wu, Xinli Wang, Fangyu Lin, Hao Chen, Yao Wang, Tao Jiang, Zhangchi Pan, Xinyan Gao, Qing Liu, Xiaojiao Weng, Na Yao, Yingjiao Zhu, Riping Wu, Guizhen Weng, Xiaoyan Lin

**Affiliations:** ^1^ Department of Oncology, Fujian Medical University Union Hospital, Fuzhou, China; ^2^ Fujian Provincial Key Laboratory of Translational Cancer Medicine, Fujian Medical University Union Hospital, Fuzhou, China; ^3^ Follow-Up Center, Fujian Medical University Union Hospital, Fuzhou, China; ^4^ Departments of Internal Medicine-Oncology, Fujian Provincial Cancer Hospital and Fujian Medical University Cancer Hospital, Fuzhou, Fujian, China; ^5^ The School of Nursing, Fujian University of Traditional Chinese Medicine, Fuzhou, China; ^6^ Department of Oncology Nursing, Fujian Medical University Union Hospital, Fuzhou, China

**Keywords:** gastric cancer, gastroesophageal junction adenocarcinoma, immune-related adverse events, anti-programmed cell death 1 receptor, prognostic prediction model, nomogram

## Abstract

**Objective:**

This study aimed to develop and validate a survival prediction model and nomogram to predict survival in patients with advanced gastric or gastroesophageal junction (G/GEJ) adenocarcinoma undergoing treatment with anti-programmed cell death 1 receptor (PD-1). This model incorporates immune-related adverse events (irAEs) alongside common clinical characteristics as predictive factors.

**Method:**

A dataset comprising 255 adult patients diagnosed with advanced G/GEJ adenocarcinoma was assembled. The irAEs affecting overall survival (OS) to a significant degree were identified and integrated as a candidate variable, together with 12 other candidate variables. These included gender, age, Eastern cooperative oncology group performance status (ECOG PS) score, tumor stage, human epidermal growth factor receptor 2 (HER2) expression status, presence of peritoneal and liver metastases, year and line of anti-PD-1 treatment, neutrophil-to-lymphocyte ratio (NLR), controlling nutritional status (CONUT) score, and Charlson comorbidity index (CCI). To mitigate timing bias related to irAEs, landmark analysis was employed. Variable selection was performed using the least absolute shrinkage and selection operator (LASSO) regression to pinpoint significant predictors, and the variance inflation factor was applied to address multicollinearity. Subsequently, a Cox regression analysis utilizing the forward likelihood ratio method was conducted to develop a survival prediction model, excluding variables that failed to satisfy the proportional hazards (PH) assumption. The model was developed using the entire dataset, then internally validated through bootstrap resampling and externally validated with a cohort from another Hospital. Furthermore, a nomogram was created to delineate the predictive model.

**Results:**

After consolidating irAEs from the skin and endocrine systems into a single protective irAE category and applying landmark analysis, variable selection was conducted for the prognostic prediction model along with other candidate variables. The finalized model comprised seven variables: ECOG PS score, tumor stage, HER2 expression status in tumor tissue, first-line anti-PD-1 treatment, peritoneal metastasis, CONUT score, and protective irAE. The overall concordance index for the model was 0.66. Calibration analysis verified the model’s accuracy in aligning predicted outcomes with actual results. Clinical decision curve analysis indicated that utilizing this model for treatment decisions could enhance the net benefit regarding 1- and 2-year survival rates for patients.

**Conclusion:**

This study developed a prognostic prediction model by integrating common clinical characteristics of irAEs and G/GEJ adenocarcinoma. This model exhibits good clinical practicality and possesses accurate predictive ability for overall survival OS in patients with advanced G/GEJ adenocarcinoma.

## 1 Introduction

Gastric or gastroesophageal junction (G/GEJ) cancer is one of the most common malignant tumors in China. Chinese G/GEJ cancer patients are characterized by late-stage diagnosis, high tumor burden, strong heterogeneity, and poor prognosis (1). Given the pronounced heterogeneity among patients with gastric cancer, accurately forecasting their survival trajectories poses substantial challenges. The current large-scale advanced gastric cancer prediction model, developed for the East Asian population based on basic clinical features, has not yet achieved optimal performance. In the study (2) by KIM et al., the area under the curve (AUC) of the one-year overall survival curve was used as a performance metric, yielding an AUC of 66.1%. Additionally, in the nomogram developed by GAO et al. (3), consistency index (C-index) was employed as the evaluation metric, with a C-index of 0.645. These metrics highlight the current model’s limitations in predicting the survival outcomes of advanced gastric cancer patients.

Currently, immune checkpoint inhibitors (ICIs) are an essential component in the treatment of G/GEJ cancer, regardless of the human epidermal growth factor receptor 2(HER2) status or the expression status of programmed cell death 1 ligand 1 (PD-L1). In the contemporary context where immunotherapy is gaining paramount importance, there is a critical demand for the development of more convenient and effective tools to assist physicians in the survival prognosis analysis of gastric cancer to formulate more individualized treatment plans.

Immune-related adverse events (irAEs) such as rash, interstitial pneumonia, hepatitis, and thyroiditis differ significantly from traditional adverse events in terms of type, mechanism, timing of occurrence, and management strategies (4). Research (5–7)indicates that patients who encounter specific irAEs may exhibit improved prognoses. Notably, gastric cancer patients experiencing irAEs demonstrate a significantly extended overall survival(OS) (8, 9). Despite these observations, no prior research has incorporated irAEs as a variable in developing a nomogram for G/GEJ cancer patients undergoing anti-PD-1 therapy to predict OS. This study was designed to employ landmark analysis to address irAEs and other clinical characteristics and construct a prognostic model and nomogram aimed at predicting survival in patients with advanced G/GEJ adenocarcinoma.

## 2 Materials and methods

### 2.1 Study population

The study was approved by the Ethics Committee of the Fujian Medical University Union Hospital(2023KY153). As it was a retrospective research design, informed consent was subsequently waived. Patients who received anti-PD-1 treatment for advanced G/GEJ cancer at our center between January 1, 2017, and December 31, 2021, were screened. The inclusion criteria were as follows: 1. Patients must be at least 18 years old at the initiation of anti-PD-1 therapy. 2. Patients must be diagnosed with gastric adenocarcinoma histologically or cytologically. 3. Patients should have undergone at least one anti-PD-1 therapy, either as monotherapy or in combination with chemotherapy. 4. At the initiation of anti-PD-1 therapy, patients must be classified as TNM stage IV or inoperable stage III. The exclusion criteria were as follows: 1. History of other malignancies; 2. pathologies other than adenocarcinoma; 3. prior or concurrent use of other ICI immunotherapies. An external validation cohort from Fujian Provincial Cancer Hospital was assembled using the same inclusion criteria.

### 2.2 Follow-up and clinical outcomes

The primary outcome of the study was OS. The follow-up period started from the first exposure to anti-PD-1 therapy and continued until the patient’s death on June 30, 2023. The research data was retrospectively collected from electronic medical records and supplemented with telephone follow-ups. For cases where the survival status was missing, the patient’s survival status was updated through telephone follow-ups between July 1, 2023, and September 30, 2023.

### 2.3 Impact of irAE on prognosis

Each irAE was first screened in the electronic medical records and then confirmed by reviewing the medical records to ensure that the AE was documented and related to ICI treatment. The irAEs were graded according to the Common Terminology Criteria for Adverse Events5.0 and categorized to the corresponding affected system for analysis. For any system with more than 10 cases, a univariate Cox analysis was performed to determine the hazard ratio (HR) and p-value. The irAE systems that met the threshold (HR less than 0.8 or greater than 1.2, and p-value less than 0.20) were then compiled separately and labeled as “beneficial irAE” or “risk irAE”. The Kaplan-Meier method was conducted on the merged variables. The variables that reached statistical significance were considered candidate variables for the construction of the prediction model.

### 2.4 Candidate variables in the predictive model

To control for bias due to the timing of irAE onset, landmark analyses (10) with a time threshold set at 60 days were performed. Only irAEs that occurred before the landmark time were considered as having occurred, while those occurring after the cutoff or not occurring at all were considered as not having occurred. Cases with death events before the landmark time are excluded. The predictive model was constructed based on the dataset processed after the landmark analyses. Other candidate clinical characteristics included in the study were gender, age, ECOG PS score, tumor stage, liver metastasis, peritoneal metastasis, tumor tissue HER2 expression status, line of anti-PD-1 treatment, year of anti-PD-1 treatment, as well as indices calculated using clinical data, including the Charlson comorbidity index (CCI), neutrophil to lymphocyte ratio (NLR), and controlling nutritional status score (CONUT) at the start of anti-PD-1 treatment. These candidate variables were transformed into binary variables. The definition of variables, missing value handling and variable transformation methods are described in **Supplementary Tables 1**–**3**.

### 2.5 Model construction and validation

The full set of variables was initially screened using least absolute shrinkage and selection operator (LASSO) regression to identify those with non-zero regression coefficients. Subsequently, variance inflation factor analysis was utilized to eliminate multicollinearity. The selected variables were then subjected to Cox regression analysis using the forward likelihood ratio method, and a predictive model was constructed by choosing clinical features significantly related to OS based on the Akaike information criterion (AIC). A nomogram was generated to explain the model.

The predictive model developed in this study was validated using both internal and external methods. Internal validation was performed employing the bootstrap resampling technique. Subsequently, an external validation cohort was utilized to further assess the model’s applicability in a separate clinical setting. Calibration curves were employed to evaluate the differences between actual observations and predicted values. The C-index was calculated and internally validated with bootstrap resampling and K-fold cross validation method. The 1-year and 2-year time-dependent receiver operating characteristic curves and time-dependent C-index curves were plotted to assess the model’s discriminative ability at different time points. Decision curve analysis (DCA) was used to evaluate clinical utility. Risk scores were calculated for each patient based on the model. The patients were divided into high-risk and low-risk groups according to the median score of all participants, and a survival analysis was performed.

### 2.6 Statistical methods

The sample size calculation was based on two conditions. First, to ascertain whether irAE influences OS, the sample size necessary for survival analysis was determined using PASS software. Reports from randomized controlled trials(RCTs) on the first-line (11) and later-line (12) ICI treatments in G/GEJ cancer patients indicated that the median OS for patients without irAE is 10 months. The HR for the group with irAE compared to the group without irAE was estimated at 0.45 (13). With an enrollment period of 60 months and a minimum follow-up of 18 months, assuming a 1:1 ratio of patients with and without irAE and a dropout rate of 10%, at least 216 patients were required under a two-sided α= 0.05 and 90% power. Second, the sample size needed for constructing a prognostic prediction model was estimated using the rule of thumb of 10 events per variable. Assuming a 75% outcome event rate and 13 candidate variables, at least 193 patients were necessary. Given these requirements, the larger value of the two conditions determined that at least 216 cases were required. Quantitative data were characterized by the median and interquartile range (IQR), whereas qualitative data were described by percentage counts. Survival analysis methods included Kaplan-Meier curves to depict survival times and Cox proportional hazards models to adjust for confounding variables. Statistical analyses were conducted using R version 4.1.5. The R package software utilized included: “survival”, “survminer”, “glmnet”, “and timeROC”. Differences were considered statistically significant when the value of *p*<0.05 in the bilateral test.

## 3 Results

### 3.1 Population

A total of 264 cases of stage III-IV G/GEJ adenocarcinoma treated with PD1 monoclonal antibody were screened. Five cases treated with ICI during the postoperative adjuvant phase were excluded. Two cases of concurrent tumors were excluded, including a case of lung cancer and a case of concurrent colon cancer. Two cases with pathological types that met the exclusion criteria were excluded, including a case of mixed glandular neuroendocrine carcinoma and a case of mesenchymal derived tumor. Lastly, 255 patients with stage III/IV G/GEJ adenocarcinoma who received anti-PD-1 inhibitor treatment were selected. The median follow-up time for the patients was 25.9 months (IQR 24–26.8 months). The average age at the start of treatment was 58.5 years, with a median age of 60 years and 67% being males. The clinical characteristics of the patients are summarized in **Table 1**.

**Table 1:** Base line clinical characteristics.

Clinical characteristics	Level	Number of patients	Percentage (%)
Age	<60	126	49.4
	≥60	129	50.6
Gender	female	84	32.9
	male	171	67.1
ECOG PS	0~1	190	74.5
	≥2	65	25.5
Stage	III	23	9.0
	IV	232	91.0
Year of anti-PD-1 Treatment	before 2021	120	47.1
	2021	135	52.9
Line of anti-PD-1 Treatment	first line	119	46.7
	second line or above	136	53.3
Her2 Expression	negative	218	85.5
	positive	37	14.5
Liver Metastasis	no	202	79.2
	yes	53	20.8
Peritoneal Metastasis	no	172	67.5
	yes	83	32.5
irAE	without irAE	119	46.7
	with irAE	136	53.3

### 3.2 Types of irAE and impact on prognosis

In this study, among the patients with G/GEJ adenocarcinoma undergoing anti-PD-1 inhibitor therapy, 136 patients experienced irAEs, representing an incidence rate of 53.5%. Out of these, 23 patients encountered grade 3–4 irAEs, which corresponds to an incidence rate of 9.0%. There were no fatalities attributed to irAEs. The distribution of irAEs across different systems is described in **Supplementary Table 3**.

The impact of irAEs on prognosis was assessed by comparing median OS and univariate Cox regression analysis. IrAEs were classified according to the system involved. The results are presented in **Table 2**.

**Table 2:** Effect of irAEs on mOS†.

Categories of irAEs	Group	No. of Patients	Median OS	(95% CI)	HR (95% CI)	*p-*value
Skin disorders	without irAE	191	8.3	(7.0–10.0)	0.77 (0.56–1.07)	0.121
	with irAE	64	12.2	(9.1–16.5)		
Endocrine disorders	without irAE	206	8	(7.0–9.3)	0.38 (0.25–0.59)	<0.001
	with irAE	49	22.6	(15.7-NA)		
Gastrointestinal disorders	without irAE	197	8.6	(8.0–11.4)	0.89 (0.63–1.26)	0.515
	with irAE	58	9.2	(6.8–15.5)		
Hepatobiliary disorders	without irAE	249	8.9	(8.1–11.4)	0.73 (0.23–2.27)	0.583
	with irAE	6	4.9	(3.8-NA)		
Musculoskeletal and connective tissue disorders	without irAE	253	9.1	(8.2–11.4)	NA	NA
	with irAE	2	5.5	(4.5-NA)		
Eye disorders	without irAE	252	8.9	(8.0–11.4)	NA	NA
	with irAE	3	15.5	(5.2-NA)		
Cardiac disorders	without irAE	253	8.9	(8.1–11.4)	NA	NA
	with irAE	2	7.4	(5.2-NA)		
Respiratory disorders	without irAE	252	8.9	(8.0–11.4)	NA	NA
	with irAE	3	9.3	(3.8-NA)		
Nervous system disorders	without irAE	254	8.9	(8.0–11.4)	NA	NA
	with irAE	1	9.5	NA		
Autoimmune disorders	without irAE	254	8.9	(8.0–11.4)	NA	NA
	with irAE	1	NA	NA		
Sepsis	without irAE	248	8.9	(8.0–11.3)	0.92 (0.41–2.08)	0.843
	with irAE	7	13.6	(4.1-NA)		
Any irAE	without irAE	119	7.2	(5.7–8.5)	0.59 (0.44–0.78)	<0.001
	with irAE	136	12.7	(9.3–15.5)		

^†^Only system disorders with more than 10 cases were analyzed with univariate Cox model.

### 3.3 Construction of the predictive model and nomogram

**Figure 1:**
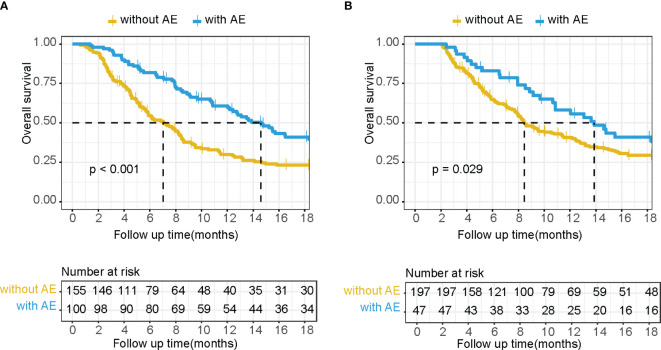
Kaplan–Meier OS curves without a landmark **(A)** and using a 2-month landmark analysis **(B)** of patients with or without beneficial irAE.

Among the systems in which irAEs occurred, only two met the preset threshold conditions for the HR and the *p*-value. These were skin disorders and endocrine disorders, both of which tended to benefit OS. These were combined into a category termed “beneficial irAE.” The median OS was significantly prolonged in patients who experienced beneficial irAE compared to those who did not (14.6 months vs. 7.0 months, *p*<0.001) (**Figure 1A**).

After excluding cases with death events before the 2-month landmark time through landmark analysis, a total of 244 cases remained. The median OS was extended in patients who experienced beneficial irAE compared to those who did not (13.9 months vs. 8.5 months, *p*=0.029) (**Figure 1B**). Beneficial irAE, along with other clinical characteristics (gender, age, ECOG PS score, tumor stage, liver metastasis, peritoneal metastasis, HER2 expression status, line of anti-PD-1 treatment, year of anti-PD-1 treatment, CCI, NLR, CONUT score), were considered as candidate variables for the predictive model.

**Figure 2:**
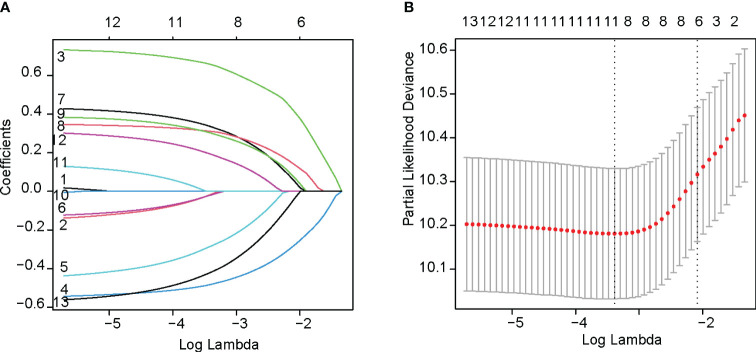
Variables selection with LASSO regression analysis. **(A)** The change of coefficients of each predictor variable according to penalty parameter(lambda). **(B)** The selection of the optimum value of the penalty parameter(lambda) with the Lasso regression.

The LASSO regression was performed using the glmnet function with the family parameter set to `cox`. After the shrinkage process, the regression coefficients of five variables were reduced to zero (**Figure 2A**). These variables included age, gender, year of anti-PD-1 treatment, liver metastasis, and CCI. **Figure 2B** illustrates how the partial likelihood deviance changes with the logarithm of the λ parameter. The lowest partial likelihood deviance is achieved when the λ value is 0.049 (indicated by the left dashed line). This study selected the λ value corresponding to the minimum partial likelihood deviance, which included 8 variables: ECOG PS score, tumor stage, tumor tissue Her2 expression status, line of anti-PD-1 treatment, peritoneal metastasis, NLR, CONUT, and beneficial irAE. Variance inflation factor analysis was conducted on these variables. All variance inflation factor values were less than 5, indicating that multicollinearity could be excluded.

**Figure 3:**
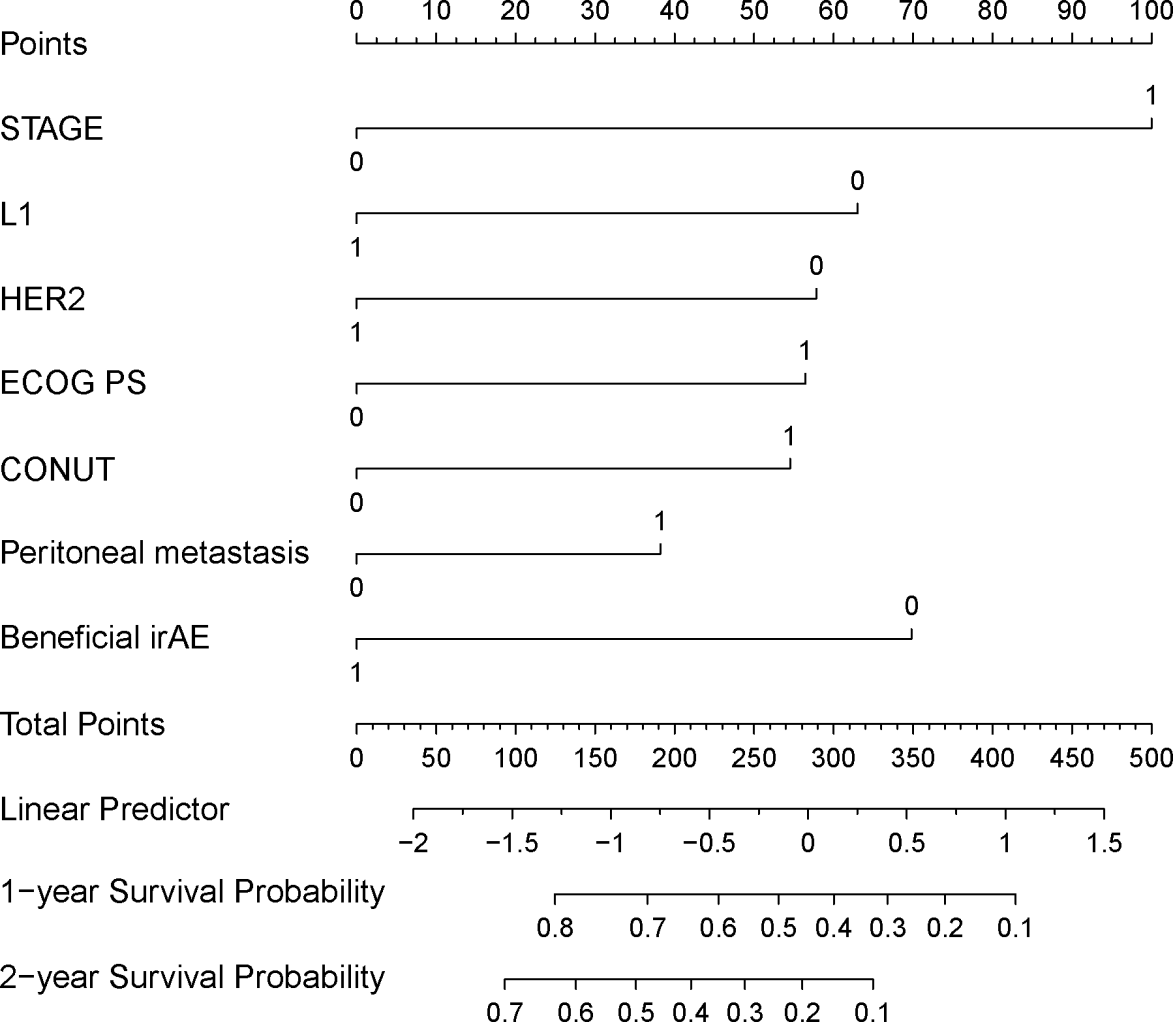
Nomogram for predicting patient 1 and 2-year survival rates. STAGE: TNM staging (stage III is 0, stage IV is 1), L1: whether the anti-PD-1 treatment was used at first line (the first line is 1, second line or above is 0); HER2: HER2 status (positive is 1, non-positive is 0); ECOG_PS: ECOG performance status score (score 0–1 is 0, score greater than or equal to 2 is 1); CONUT: Control nutritional status score (CONUT score less than or equal to 6 is 0, greater than 6 is 1); Peritoneal_metastasis: Whether there is peritoneal metastasis (1 if there is metastasis, 0 if there is no metastasis); Beneficial_irAE: Whether a beneficial irAE occurs within 2 months (occurrence is 1, otherwise it is 0).

The variables selected from the LASSO regression were subjected to Cox regression analysis using the Forward Likelihood Ratio method to identify clinical features significantly related to OS for inclusion in the predictive model. After the forward likelihood ratio method Cox regression analysis, all eight variables were retained. When performing the proportional hazards assumption test on all eight variables, it was found that NLR failed the proportional hazards assumption test. Consequently, NLR was removed from the model. Univariate and multivariate Cox regression analyses were then conducted on the remaining 7 variables (**Table 3**). The AIC for the model, which included all seven variables, was 1676.578. The AIC for the model excluding HER2 was1679.295. The AIC for the model excluding peritoneal metastasis was 1678.024. Following the principle of minimizing AIC, both HER2 and peritoneal metastasis were included as variables in the final prognostic prediction model. The final prediction model included 7 variables: ECOG PS score, tumor stage, tumor tissue Her2 expression status, anti-PD-1 treatment lines, peritoneal metastasis, CONUT, and beneficial irAE. A nomogram was constructed based on the model (**Figure 3**), which can be used to predict 1- and 2-year survival rate for patients with advanced G/GEJ adenocarcinoma.

**Table 3:** Cox regression **analysis** results of each variable included in the model.

Variable	Univariable Cox regression		Multivariable Cox regression	
	Crude HR (95% CI)	*p*-value	Adjusted HR (95% CI)	*p*-value
Stage	3.55 (1.80–6.99)	<0.001	2.24(1.10 - 4.54)	0.025
Line of Treatment	0.49 (0.36–0.66)	<0.001	0.60(0.44 - 0.83)	0.002
HER2	0.68 (0.44–1.05)	0.079	0.63(0.40 - 0.98)	0.040
ECOG PS	1.73 (1.25–2.41)	0.001	1.58(1.12 - 2.21)	0.008
COUNT	1.72 (1.28–2.32)	<0.001	1.55(1.14 - 2.12)	0.005
Peritoneal Metastasis	1.55 (1.14–2.11)	0.005	1.36(0.99 - 1.88)	0.060
Beneficial irAEs	0.65 (0.44–0.96)	0.031	0.57(0.38 - 0.85)	0.006

### 3.4 Model validation

**Figure 4:**
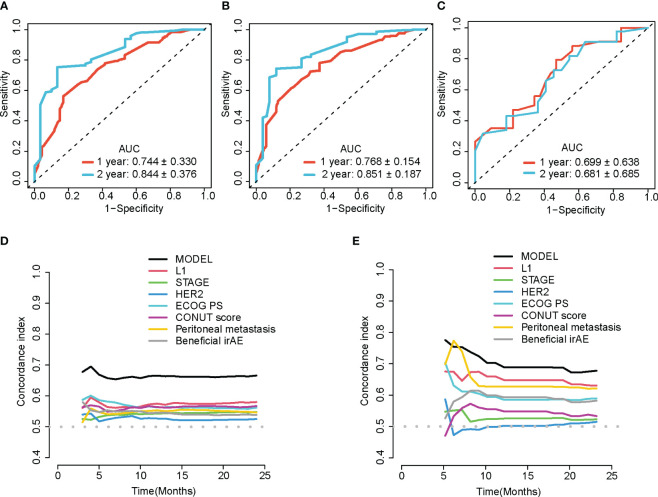
Time-dependent ROC and C-index curves. Time-ROC curves at 1- and 2-year time points of the training set **(A)**, the internal validation set **(B)** generated by bootstrap 1000 resampling and the external validation set **(C)**. Time-dependent C-index curves show the concordance index of the model and each variables calculated based on the training set **(D)** or external validation set **(E)**. MODEL, the prediction model; STAGE, TNM stage; L1, first-line treatment; HER2, HER2 status; ECOG PS, ECOG performance status score; CONUT, control nutritional status score.

An external validation cohort consisting of 66 patients from Fujian Provincial Cancer Hospital was assembled. The time dependent receiver operating characteristic(time-ROC) curve of the model was plotted based on the training dataset, the internal validation dataset from 1000 bootstrap samples and the external validation dataset (**Figures 4A–C**).

The model’s C-index was 0.66. Based on 1000 bootstrap resampling method with the bias-corrected interval approach, the 95% confidence interval for the C-index was (0.61–0.70). Furthermore, 5-fold cross-validation yielded an internal validation C-index of 0.65(95%CI: 0.647–0.654) and the C-index for the external validation was 0.64. The comparison of the C-index of the model with that of individual clinical indicator variables in the original dataset (**Figure 4D**) and the external validation set (**Figure 4E**) showed that the model’s discriminatory power was superior to the performance of each individual variable over two years.

**Figure 5:**
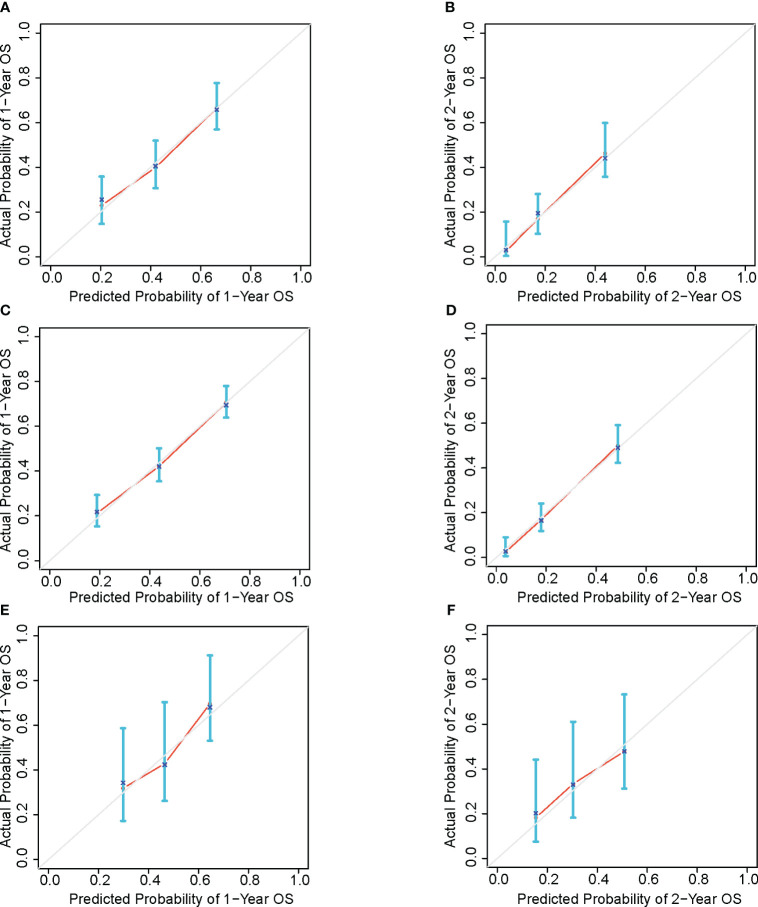
OS calibration curve based on training set of 1-year **(A)** and 2-year **(B)**, bootstrap resampling dataset of 1-year **(C)** and 2-year **(D)** and external validation set of 1-year **(E)** and 2-year **(F)**.

The calibration curves for the 1- and 2-year survival rates based on the training dataset, the internal validation dataset and the external validation dataset were plotted (**Figure 5**), showing an acceptable consistency between the predicted results and the actual observations.

**Figure 6:**
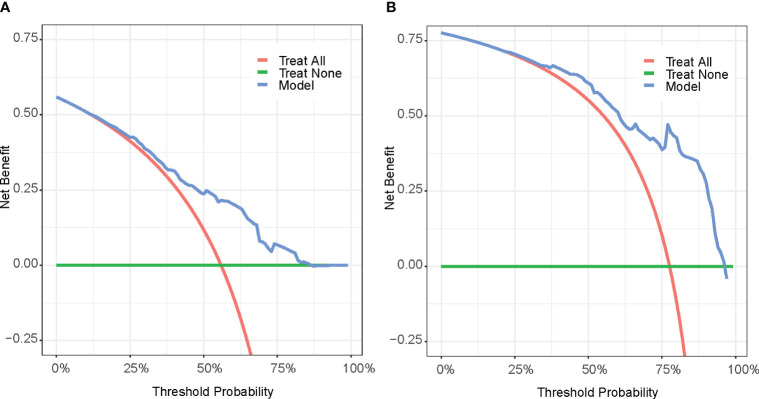
Decision curve analysis (DCA) of 1-year survival **(A)** and 2-year survival **(B)**. Treat All (red line): Treat all patients; Treat None (green line): Do not treat any patients. Model(blue line): Decision curve according to the Model.

The DCA curves (**Figure 6**) were used to evaluate the net benefit provided by our prognostic prediction model in clinical decision-making scenarios. For patients who experienced death within one year, our model offers a net benefit over a strategy of either treating all patients or not treating any patient (represented by the probability threshold range of approximately 20%–85%). For those who died within two years, the model provides a net benefit for a probability threshold range of about 32%–94% compared to a strategy where all patients are treated, or none are treated.

Based on the constructed model, the risk score for each patient was calculated according to the following formula:


$$\begin{array}{c} \text{risk score}=0.455\times \left(\text{ECOG PS}\right)+0.807\times \text{stage}-0.466\\ \times \left(\text{Her}2\text{ expression}\right)-0.508\times \left(\text{anti}-\text{PD}-1\text{ treatment line}\right)\\ +0.308\times (\text{peritonealmetastasis})\\ +0.439\times \text{CONUT}-0.562\times \left(\text{beneficial irAE}\right) \end{array}$$

The patients were divided into high- and low-risk groups based on the median risk score. KM analysis showed the differences in survival between the high- and low-risk groups. There was a significant difference in median OS between the two groups, with values of 7.0 (95%CI 5.2–8.4) months versus 15.7(95%CI 13.1–26.0) months, respectively, and HR was 0.35(95%CI 0.25–0.47).

## 4 Discussion

In this study, we developed a prognostic prediction model for patients with advanced G/GEJ adenocarcinoma treated with PD1 monoclonal antibodies. The model’s C-index stands at 0.66(95%CI 0.61–0.70), significantly outperforming individual variables in overall discriminability. Most existing research on prognostic models for advanced G/GEJ cancer (2, 3, 14) does not focus on populations undergoing ICI therapy. To the best of our knowledge, this is the first nomogram that incorporates irAE as a predictive factor for forecasting the prognosis of patients with advanced G/GEJ adenocarcinoma receiving ICI treatment.

The results of the univariate and multivariate Cox regression analyses are presented in **Table 3**. A notable change in the significance level is observed for the variable peritoneal metastasis between the univariate (p=0.005) and multivariate (p=0.060) Cox regression analyses. This shift may be due to interactions between peritoneal metastasis and other variables, such as the inevitable staging of peritoneal metastasis as stage IV. Peritoneal metastasis could also be associated with varying degrees of intestinal dysfunction, leading to a poorer nutritional status and diminished physical function. Therefore, after adjusting for multiple factors in the Cox analysis, the initially prominent significance of the peritoneal metastasis variable in the univariate analysis diminished to non-significance. Concurrently, the HER2 status shifted from non-significant (univariate p=0.079) to significant (multivariate p=0.040). Research (15) has indicated that the absence of peritoneal metastasis correlated independently with HER2 positivity. Consequently, it appeared that while the prognostic influence of peritoneal metastasis diminished following multivariate Cox adjustment, the prognostic trend of HER2 positivity became more pronounced.

Incorporating irAE as a candidate variable in the prognostic prediction model revealed a significant influence of irAE occurrence on prognosis. This study observed that cases with irAE exhibited a median OS that surpassed that of those without irAE (**Table 2**). Subgroup analysis indicated variable impacts of different types of irAE on prognosis. Drawing on the work (13, 16) of other researchers, we grouped the endocrine and skin system irAEs into a single variable named “beneficial irAE,” which was then utilized in constructing the prognostic prediction model.

The prognostic factor “beneficial irAE” had a p-value in the multivariate Cox regression analysis that was an order of magnitude lower than the p-value in the univariate Cox regression analysis, thus becoming more significant. This result indicated that after fully accounting for the influence of other variables, “beneficial irAE” had a significant effect on survival time. Future research might need to further explore the biological mechanisms linking irAE and survival time, offering a deeper and more comprehensive understanding of how variables impact survival time.

Our model included HER2, a significant molecular pathological marker of G/GEJ adenocarcinoma. In this study, multivariate Cox analysis revealed that HER2 positivity serves as a protective factor for patients with advanced G/GEJ adenocarcinoma undergoing anti-PD-1 therapy. The role of HER2 status in the prognosis of G/GEJ adenocarcinoma remains debated. On the one hand, some studies suggest that HER2 overexpression (17, 18) is correlated with a poor prognosis. On the other hand, patients with HER2 positivity who undergo HER2-targeted therapy tend to have a more favorable prognosis (19) than those who are HER2 negative(HR=0.58, 95%CI 0.36–0.95, P = 0.03). In the current era of immunotherapy, the impact of HER2 positivity on prognosis still needs clarification. Currently, large RCTs (20–23) on ICI treatment for G/GEJ adenocarcinoma have not yet provided stratified analysis based on HER2 status. We hypothesized that among patients with advanced G/GEJ adenocarcinoma, those with HER2 positivity might exhibit a better prognosis when treated with a combination of ICI, chemotherapy, and targeted HER2 therapy compared to HER2-negative patients treated with a combination of ICI and chemotherapy alone. This hypothesis requires further investigation.

Currently, model research targeting the G/GEJ adenocarcinoma population treated with ICIs often incorporates complex predictive variables. For example, expression of specific genes (24, 25) or special test indicators (26, 27) such as interleukins and CD4^+^/CD8^+^ lymphocytes. Real-world retrospective data often lacks these conditions. Large sample retrospective G/GEJ adenocarcinoma prognostic prediction models commonly use datasets from the SEER database. The SEER database has shortcomings, such as incomplete medication records and lack of adverse reaction records. Immunotherapy has currently become a major treatment for G/GEJ adenocarcinoma. Incorporating irAE as one of the predictors in the constructed model may more accurately predict patient outcomes, given the established correlation between irAE and prognosis. The types of irAEs included in our study were based on monitoring indicators recommended by major guidelines, which do not rely on special indicators like genomics and do not increase the additional financial burden on patients. Therefore, this model has good clinical practicality.

Considering that the efficacy of ICIs is influenced by numerous factors, the model in this study also incorporated indicators across multiple dimensions, including the inflammatory marker NLR, the nutritional index CONUT, and the patient’s underlying disease index CCI. NLR may reflect the body’s inflammatory response to tumors and is a predictive factor (28)for G/GEJ adenocarcinoma, as well as considered a predictive factor for ICI treatment in several cancer types (29, 30). Studies have found that the CONUT score, as a nutritional indicator, can predict survival in patients with gastric cancer (31) and esophageal cancer (32) undergoing ICI. CCI can serve as survival predictors for patients (33) with gastric adenocarcinoma. Therefore, this study collected and calculated the above indicator variables from clinical data.

Landmark analysis helps control biases (34) stemming from the different times irAEs occur among patients. By categorizing patients based on whether they experienced irAEs before or after a specified landmark time, we made the model more interpretable and enhanced its acceptance and practical value in clinical settings. Large-sample studies (11, 35) commonly use six months as the landmark time to assess the impact of irAEs on prognosis, as this period typically captures the majority of irAE occurrences noted in these studies. However, we selected a 2-month timepoint for our landmark analysis for several reasons. First, the median occurrence time for irAEs in our study and others (36) is around 2 months post-immunotherapy initiation (**Table 2**), ensuring a substantial proportion of cases are included at this early stage. Second, given the short median OS for G/GEJ adenocarcinoma patients—ranging from about 6–18 months in RCTs (12, 21)—a prediction made at an earlier point is more valuable. If the 6-month mark had been chosen, many patients might have already succumbed, making the predictions less relevant. Lastly, G/GEJ cancer often presents with lesions that are challenging to accurately assess for progression, such as those in the gastric wall, ascites, and peritoneal metastases. In clinical practice, the first efficacy assessment typically occurs around 6–8 weeks. Our model, therefore, serves as a timely tool during this initial evaluation period, aiding clinicians in prognostic assessments for G/GEJ cancer patients.

Although our model provided valuable prognostic information, it has some limitations. First, our study sample was drawn from a single medical center and lacked external validation of large populations or different regions, which could limit the general applicability of the results. Second, because the study was retrospective and relied on existing medical records, some data were missing. Laboratory information was insufficient for certain cases with rare irAEs, and the selection of variables could not have been comprehensive. Our model did not include PDL1 expression and the mismatch repair status because the study found that cases untested for these two indicators accounted for more than 30% of all cases. In the real world, the detection rates of PDL1 expression and mismatch repair status in G/GEJ adenocarcinoma patients are much lower than those in RCTs with strict enrollment criteria, possibly due to reasons such as limited biopsy specimen slices not meeting the needs of multiple immunohistochemical tests, and resistance to re-biopsy by patients. The absence of these biomarker variables, which affect the efficacy of immunotherapy, can limit the performance of the model.

## 5 Conclusions

This study utilized the landmark method to control time-dependent bias and constructed a prognostic prediction model for G/GEJ adenocarcinoma under anti-PD-1 therapy using irAEs and common clinical characteristics as predictors. The model can predict patient OS with moderate accuracy. Future studies could improve the model by incorporating additional variables and employing multicenter data for validation.

## Data availability statement

The raw data supporting the conclusions of this article will be made available by the authors, without undue reservation.

## Ethics statement

The studies involving humans were approved by the Ethics Committee of the Fujian Medical University Union Hospital. The studies were conducted in accordance with the local legislation and institutional requirements. The ethics committee/institutional review board waived the requirement of written informed consent for participation from the participants or the participants’ legal guardians/next of kin because the study was a retrospective research design.

## Author contributions

HW: Writing – original draft, Writing – review & editing. JC: Writing – review & editing. WG: Data curation, Validation, Writing – review & editing. YLW: Writing – original draft. XLW: Writing – original draft. FL: Writing – original draft. HC: Writing – original draft. YW: Writing – original draft. TJ: Writing – original draft. ZP: Writing – original draft. XG: Writing – original draft. QL: Writing – original draft. XJW: Writing – original draft. NY: Writing – original draft. YZ: Writing – original draft. RW: Writing – original draft. GW: Writing – original draft. XL: Writing – review & editing.
