# Supplementary material for: Construction of a nomogram with IrAE and clinic character to predict the survival of advanced G/GEJ adenocarcinoma patients undergoing anti-PD-1 treatment
Source: Front Immunol. 2024 Jul 24;15:1432281. doi: 10.3389/fimmu.2024.1432281 (PMC11303212; doi:10.3389/fimmu.2024.1432281)
Supplement: Supplementary file 1 [file Table_1.docx]

Supplementary Material

# Supplementary Table1. Definition of some complex variables

| Variable | Variable definition |
| --- | --- |
| CCI | The Charlson Comorbidity Index (CCI)(1) is a weighted score used to evaluate the comorbidities of patients. It includes 19 different disease categories, each of which is assigned a weight based on its impact on patient prognosis. The total score is the sum of the weights of each category, ranging from 0 to 33, with higher scores indicating more severe comorbidities. |
| CONUT | The Controlling Nutritional Status (CONUT) score, first proposed by Ignacio (2), is a method used to evaluate the nutritional status of patients. Based on three laboratory biomarkers: serum albumin, total lymphocyte count, and total cholesterol, the CONUT score was calculated by evaluating the three indicators. The higher the CONUT score, the poorer the patient's nutritional status. |
| Her2 Expression | HER2 status is divided into positive and non positive. Positive is defined as immunohistochemical staining 3+or immunohistochemical staining 2+with positive fluorescence in situ hybridization. Non positive includes two situations: the test result is not positive and not tested. |
| NLR | The Neutrophil to Lymphocyte Ratio (NLR) refers to the ratio of neutrophil to lymphocyte values in peripheral blood. |
| Peritoneal Metastasis | Peritoneal Metastasis encompasses metastases to the parietal peritoneum, visceral peritoneum, mesentery, greater omentum, and other organ surfaces within the abdominal cavity. The criteria for confirming peritoneal metastasis include: identification of metastasis through imaging reports, direct visual observation during surgical exploration, or the detection of tumor cells in ascites cytology. |

# Supplementary Table2. Missing values and imputation methods

| Variable | Variable type | Missing cases | Percent of missing | Method of imputation |
| --- | --- | --- | --- | --- |
| Her2 Expression | binary | 48 | 18.8 | imputed with negative† |
| CONUT | ordered categorical | 19 | 7.5 | imputed with 0†† |
| NLR | continuous | 11 | 4.3 | imputed with median |
| Age | continuous | none | NA |  |
| Gender | binary | none | NA |  |
| ECOG PS | ordered categorical | none | NA |  |
| Stage | binary | none | NA |  |
| Year of Anti PD-1 Treatment | ordered categorical | none | NA |  |
| First Line Anti PD-1 Treatment | binary | none | NA |  |
| Liver Metastasis | binary | none | NA |  |
| Peritoneal Metastasis | binary | none | NA |  |
| CCI | ordered categorical | none | NA |  |
| Benificial irAE | binary | none | NA |  |

†HER2 positive patients who did not receive treatment with trastuzumab had similar survival rates to HER2 negative patients (3) (HR1.04; 95% CI 0.52-2.11; P=0.91)

††Biochemical function and blood routine test results are required to be within normal range before anti-tumor treatment. Cases with missing laboratory results in our unit are likely to have laboratory tests conducted in other hospitals with normal results. The CONUT score calculated based on normal values is 0.

# Supplementary Table3. Method of converting variables into binary variables

| Variable | Variable type | Method of converting |
| --- | --- | --- |
| Age | continuous | Divided according to the median |
| NLR | continuous | Divided according to the best cutoff† |
| ECOG PS | ordered categorical | Divided by ＜2 or ≥2 |
| Year of Anti PD-1 Treatment | ordered categorical | Divided by before 2021 or 2021 |
| CCI | ordered categorical | Divided according to the best cutoff† |
| CONUT | ordered categorical | Divided according to the best cutoff† |

† To Find the best cutoff value, the surv_cutton function in the R package "survminer" was used.

# Supplementary Table4. Distribution of irAEs across different systems

| Categories of irAEs | Any grade | Grade 3-4 | Median Time to irAE(days) | IQR |
| --- | --- | --- | --- | --- |
| **Skin disorders** | 64 | 2 | 64 | (44-102) |
| rash | 54 | 2 | 59 | (44-97) |
| pruritus | 15 | 0 | 65 | (42-136) |
| Telangiectasia or Reactive cutaneous capillary endothelial proliferation | 4 | 0 | 282 | (215-345) |
| **endocrine disorders** | 49 | 3 | 75 | (32-170) |
| Abnormal thyroid function | 43 | 1 | 75 | (35-165) |
| Cortisol reduction | 5 | 1 | 28 | (21-243) |
| Pituitary inflammation | 3 | 1 | 273 | (148-350) |
| **Gastrointestinal disorders** | 58 | 6 | 104 | (35-197) |
| Oral mucosal inflammation | 5 | 0 | 32 | (22-120) |
| Enteritis/Diarrhea | 48 | 2 | 116 | (43-228) |
| pancreatitis | 9 | 4 | 57 | (31-197) |
| **Hepatobiliary disorders** | 6 | 5 | 76 | (64-83) |
| Immunological hepatitis | 6 | 4 | 76 | (64-83) |
| Immune cholangitis | 1 | 1 | 152 | NA |
| **Musculoskeletal disorders** | 2 | 0 | 76 | (66-87) |
| Arthritis | 1 | 0 | 98 | NA |
| Myositis | 1 | 0 | 55 | NA |
| **vision disorders** | 3 | 0 | 172 | (128-282) |
| conjunctivitis | 3 | 0 | 172 | (128-282) |
| **Cardiac disorders** | 2 | 2 | 52 | (37-68) |
| autoimmune myocarditis | 2 | 2 | 52 | (37-68) |
| **respiratory disorders** | 3 | 3 | 82 | (80-118) |
| Immune pneumonia | 3 | 3 | 82 | (80-118) |
| **nervous disorders** | 1 | 1 | 22 | NA |
| myasthenia gravis | 1 | 1 | 22 | NA |
| **Autoimmune disorders** | 1 | 0 | 391 | NA |
| Sjogren's syndrome | 1 | 0 | 391 | NA |
| **sepsis** | 7 | 7 | 208 | (138-400) |

# References of supplementary material

1. Charlson ME, Pompei P, Ales KL, MacKenzie CR. A new method of classifying prognostic comorbidity in longitudinal studies: development and validation. Journal of chronic diseases. 1987;40(5):373-83.

2. Ignacio de Ulibarri J, Gonzalez-Madrono A, de Villar NG, Gonzalez P, Gonzalez B, Mancha A, et al. CONUT: a tool for controlling nutritional status. First validation in a hospital population. Nutricion hospitalaria. 2005;20(1):38-45.

3. Shitara K, Yatabe Y, Matsuo K, Sugano M, Kondo C, Takahari D, et al. Prognosis of Patients with Advanced Gastric Cancer by Her2 Status and Trastuzumab Treatment. Gastric Cancer (2013) 16(2):261-7. doi: 10.1007/s10120-012-0179-9.
